# EEG-Driven Arm Movement Decoding: Combining Connectivity and Amplitude Features for Enhanced Brain–Computer Interface Performance

**DOI:** 10.3390/bioengineering12060614

**Published:** 2025-06-04

**Authors:** Hamidreza Darvishi, Ahmadreza Mohammadi, Mohammad Hossein Maghami, Meysam Sadeghi, Mohamad Sawan

**Affiliations:** 1 Department of Cognitive Psychology, Institute for Cognitive Science Studies (ICSS), Tehran 16583-44575, Iran; darvishi_hr@icss.ac.ir (H.D.); mohammadi_a@icss.ac.ir (A.M.); m.sadeghi@icss.ac.ir (M.S.); 2 Research Laboratory for Integrated Circuits, Faculty of Electrical Engineering, Shahid Rajaee Teacher Training University, Tehran 16788-15811, Iran; 3 Center of Excellence in Biomedical Research on Advanced Integrated-on-Chips Neurotechnologies (CenBRAIN Neurotech), School of Engineering, Westlake University, Hangzhou 310030, China

**Keywords:** brain–computer interface, electroencephalography, electromyography, filter bank common spatial patterns, phase-locking value, neural network, feature selection, ReliefF, movement decoding

## Abstract

Brain–computer interfaces (BCIs) translate electroencephalography (EEG) signals into control commands, offering potential solutions for motor-impaired individuals. While traditional BCI studies often focus solely on amplitude variations or inter-channel connectivity, movement-related brain activity is inherently dynamic, involving interactions across regions and frequency bands. We propose that combining amplitude-based (filter bank common spatial patterns, FBCSP) and phase-based connectivity features (phase-locking value, PLV) improves decoding accuracy. EEG signals from ten healthy subjects were recorded during arm movements, with electromyography (EMG) as ground truth. After preprocessing (resampling, normalization, bandpass filtering), FBCSP and multi-lag PLV features were fused, and the ReliefF algorithm selected the most informative subset. A feedforward neural network achieved average metrics of: Pearson correlation 0.829 ± 0.077, R-squared value 0.675 ± 0.126, and root mean square error (RMSE) 0.579 ± 0.098 in predicting EMG amplitudes indicative of arm movement angles. Analysis highlighted contributions from both FBCSP and PLV, particularly in the 4–8 Hz and 24–28 Hz bands. This fusion approach, augmented by data-driven feature selection, significantly enhances movement decoding accuracy, advancing robust neuroprosthetic control systems.

## 1. Introduction

**Figure 1:**
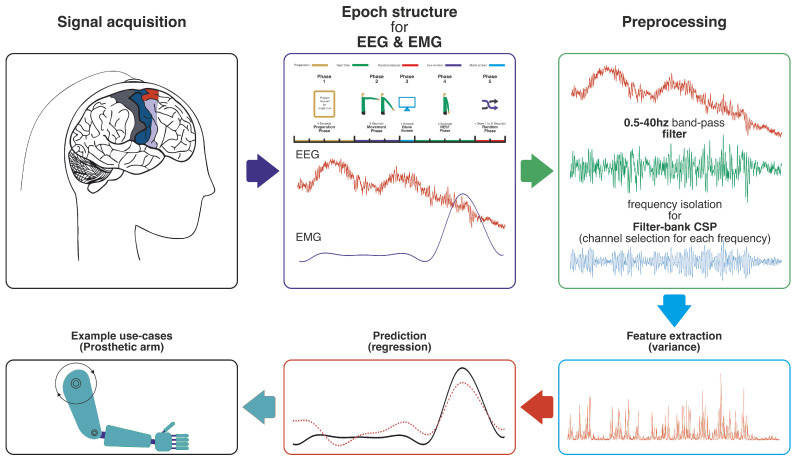
A brain–computer interface pipeline (signal variance is illustrated as an extracted features example). The pipeline typically involves EEG signal acquisition, preprocessing to remove noise and artifacts, extraction of relevant neural features (such as amplitude modulations or connectivity patterns), and a decoding model (e.g., a machine-learning algorithm) that translates these features into control commands or predicted movement parameters, as explored in this work for EEG-to-EMG regression.

BCIs offer significant potential for individuals with severe motor disabilities by translating brain signals directly into control commands [1]. Early BCIs primarily enabled basic communication by classifying discrete brain states, such as steady state evoked potentials (SSVEP) [2]. However, the goal of controlling complex devices like prosthetic limbs with natural fluidity necessitates a shift towards continuously decoding the rich tapestry of motor intentions. While invasive methods have demonstrated high fidelity in continuous decoding, including intracortical microelectrode recordings [3,4,5] and electrocorticography (ECoG) which has shown comparable accuracy for decoding movement trajectories [6,7,8], their clinical application is limited by surgical risks and long-term stability concerns [9]. Consequently, non-invasive EEG has gained prominence due to its safety and practicality, despite the inherent challenges of extracting clear motor commands from its relatively noisy signals (a general EEG-BCI pipeline, similar to our decoding approach, is illustrated in Figure 1).

The endeavor to decode precise motor control from EEG has been multifaceted. Traditional approaches often focused on amplitude modulations within specific frequency bands, such as event-related desynchronization in mu/beta rhythms [10], or information in very low-frequency components. For instance, Mondini et al. (2020) achieved moderate correlations (≈0.32–0.5) for online robotic arm control using EEG signals filtered between 0.18–1.5 Hz [11]. However, these foundational strategies may not fully capture the brain’s intricate coordination during movement. Recognizing this, some researchers turned to deep learning. For instance, models like interpretable convolutional neural networks (ICNNs) have been used to decode 2D hand kinematics from broadly filtered EEG [12]. Other approaches have employed techniques such as multi-branch CNNs with attention mechanisms [13], Transformer-based architectures designed to capture global signal dependencies [14], or hybrid models combining CNNs and LSTMs with residual connections for motor imagery classification [15]. While powerful, such end-to-end models often learn features implicitly, contrasting with methods that explicitly engineer and combine neurophysiologically distinct signatures, potentially offering a more targeted understanding of underlying neural processes.

The pursuit of a more comprehensive representation of neural dynamics has also led to exploring functional connectivity and advanced decoding paradigms. Hosseini and Shalchyan (2022) investigated phase-based connectivity using PLV and magnitude-squared coherence (MSC)—a technique also explored for detecting event-related activity during motor imagery, particularly in very low-frequency bands [16]—from predominantly delta and alpha EEG bands with multiple linear regression to decode 2D hand position. They achieved Pearson correlations of R≈ 0.42–0.43 and demonstrated superiority over amplitude-only methods in their center-out task [17]. Building on this, their 2023 work introduced a state-based approach: a discrete classifier using common spatial patterns (CSP) from pre-movement EEG first identified the movement axis (97.1% binary accuracy), followed by separate Gaussian process regression (GPR) models using EEG envelope features for continuous trajectory decoding along each axis. This hybrid strategy improved kinematic prediction, yielding R=0.54 for principal targets and generalizing to random targets (R=0.37) [18]. Alongside these methodological advancements, the drive for real-world applicability has seen studies validating motor decoding with commercial mobile EEG systems. For example, Robinson et al. (2021) used an Emotiv EPOC headset and FBCSP features from low-frequency EEG (primarily < 5.5 Hz) for hand speed classification, successfully reconstructing 1D hand position and speed with correlations from R≈ 0.22–0.58 [19]. The exploration of neural dynamics underlying motor tasks has also involved sophisticated analyses; for instance, EEG microstate-specific functional connectivity (using measures like PLV) has been employed to differentiate motor states such as motor execution, imagery, and visually guided imagery, aiming to provide insights for BCI rehabilitation strategies [20]. These diverse efforts highlight the ongoing search for robust features and paradigms to enhance non-invasive decoding, moving beyond single-modality features or simple linear models.

A particularly promising avenue for enhancing neuroprosthetic control is the direct regression of EMG signals from EEG. EMG reflects muscle activation and offers intuitive control, especially for users with insufficient residual muscle activity for conventional myoelectric prostheses [21], as it encodes rich information about force, timing, and muscle coordination crucial for dexterous prosthetic function. Liang et al. (2020) made significant strides in EEG-to-EMG decoding, proposing a “virtual flexor-extensor muscle” concept to estimate shoulder EMG from 7-channel EEG (0.1–45 Hz band). They utilized independent component analysis (ICA) for artifact rejection, short-time Fourier transform (STFT) for spectral features, and principal component analysis (PCA) for dimensionality reduction, feeding these features into a linear model. Their approach achieved an average Pearson correlation of R=0.78 between actual and estimated deltoid EMG, demonstrating potential for EEG-driven power augmentation in exoskeletons [22]. While this work provides a strong precedent, and other research has explored combined EEG-EMG for rehabilitation robots [23], there remains an opportunity to further enhance EEG-to-EMG decoding. Specifically, the systematic investigation of explicitly fusing broadband amplitude-based features (like FBCSP) with phase-based connectivity features (like multi-lag PLV), processed by a non-linear model (like a neural network), has not been fully explored for EMG prediction. Many connectivity studies used linear models or focused on kinematic targets (e.g., [17]), and the state-based approach in [18], while effective for kinematics, relied on amplitude envelope features for its continuous decoding stage rather than incorporating phase connectivity.

This study aims to bridge this gap. We hypothesize that explicitly fusing information from broadband amplitude-based features (FBCSP) and phase-based connectivity features (PLV), followed by data-driven feature selection, will improve EMG signal decoding accuracy compared to approaches relying on either feature type in isolation or features from restricted frequency ranges. We extract amplitude information using FBCSP across multiple frequency bands and phase synchronization using multi-lag PLV to capture instantaneous and delayed interactions. The ReliefF algorithm selects features from the combined pool, and a feedforward neural network predicts EMG amplitudes. This integrated methodology is designed to capture a more comprehensive representation of neural dynamics underlying voluntary movement, aiming to advance accurate and reliable BCI control for neuroprosthetic applications by directly targeting peripheral muscle activity.

## 2. Materials and Methods

This section details the experimental setup, data acquisition, preprocessing techniques, feature extraction methods, and the neural network model used to decode arm movement parameters from EEG and EMG signals. The individual steps of this pipeline correspond to the stages labeled in Figure 1.

### 2.1. Experimental Paradigm

**Figure 2:**
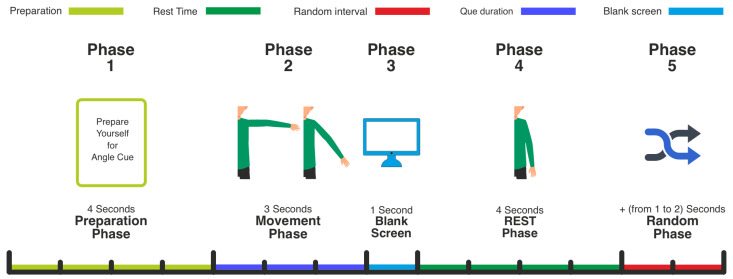
Schematic of the experimental paradigm and trial timing. Each trial consists of a 4-s preparation phase, a 3-s movement phase, and a 4-s rest phase with a random inter-trial interval of 1 to 2 s.

Each trial comprised preparation, movement, and rest phases (Figure 2). The sequence was:

1. Preparation Phase (4 s): At trial onset, a 4-s preparatory cue prompted participants to prepare for the movement, ensuring consistent readiness.
2. Movement Phase (3 s): Following preparation, a 3-s movement cue indicated one of the predefined hand angles via distinct hand images. The cued angles corresponded to three distinct levels of right arm abduction in the horizontal plane (e.g., low, middle, high elevation), primarily engaging the deltoid muscle. Participants positioned their right hand at the indicated angle upon cue presentation.
3. Blank Screen (1 s): After movement, a 1-s blank screen indicated the trial’s end, and participants lowered their hand to a neutral position.
4. Rest Phase (4 s): A 4-s rest period ensued, allowing participants to return their hand to the central origin.
5. Random Inter-Trial Interval (1–2 s): The rest phase was prolonged by a random interval (1–2 s) to ensure trial timing variability.

#### 2.1.1. Calibration Phase

Prior to experimental trials, a calibration phase established baseline EMG activity for each movement angle. Participants performed arm lifts at the three designated abduction levels for 5 s each, across 15 trials (5 trials per angle level). This facilitated accurate mapping of EMG signals to specific arm movements, enhancing regression analysis reliability.

#### 2.1.2. Data Synchronization

EEG and EMG data were simultaneously recorded on an amplifier to maintain precise synchronization between brain activity and muscle movements. This synchronization was essential for training the neural network to predict EMG amplitudes from EEG features, ensuring the model could accurately relate brain activity to muscle activation.

#### 2.1.3. Participant Instructions

Participants received comprehensive instructions on task objectives and trial procedures. They were trained to respond promptly to movement cues, maintain consistent movement amplitudes corresponding to the indicated abduction levels, minimize extraneous movements, and adhere to timing specifications to ensure data integrity.

#### 2.1.4. Ethical Compliance

This study was conducted in accordance with the Declaration of Helsinki, and the protocol was approved by the Research Ethics Committee, Institute for Cognitive Science Studies (Approval Code: IR.UT.IRICSS.REC.1403.001) on 16 April 2024.

### 2.2. Data Preprocessing

Raw EEG and EMG recordings underwent preprocessing to optimize subsequent feature extraction and analysis. These steps enhanced signal quality by eliminating artifacts, normalizing data, and preparing signals for feature extraction. Given EEG’s susceptibility to noise (e.g., ocular, muscular artifacts) and EMG’s vulnerability to baseline fluctuations and motion interference, appropriate filtering, artifact removal, and normalization were critical for clean data representative of underlying neural and muscular activity.

#### 2.2.1. EEG Preprocessing

Raw EEG data were preprocessed to eliminate artifacts, improve signal-to-noise ratio, and prepare signals for feature extraction.

1. Filtering:EEG data were processed using a zero-phase, fourth-order Butterworth filter with a 0.1–40 Hz passband.

(1) $$H\left(s\right)=\frac{1}{1+{\left(\frac{s}{\omega_{c,high}}\right)}^{2n}}\cdot \frac{1}{1+{\left(\frac{\omega_{c,low}}{s}\right)}^{2n}}$$

where $\omega_{c,high}=2\pi \times 40$ rad/s, $\omega_{c,low}=2\pi \times 0.1$ rad/s, and n=4 is the filter order. The 0.1 Hz high-pass cutoff removed slow drifts. The 40 Hz low-pass cutoff retained relevant motor activity components (delta through low gamma) while reducing high-frequency noise and muscle artifacts. A zero-phase filter prevented phase distortions. A separate notch filter was not employed, as the bandpass filter effectively addressed powerline noise.

2. Artifact Removal: Independent Component Analysis (ICA) [24], a blind source separation method, was applied to decompose multi-channel EEG data into statistically independent components, separating neural signals from artifacts like ocular, muscular, or electrode movements.

(2) $$r=\frac{\mathrm{cov}(S_{i},\mathrm{EOG})}{\sigma_{S_{i}}\sigma_{\mathrm{EOG}}}>0.4$$

Components exhibiting a Pearson correlation (r > 0.4) with simultaneously recorded electrooculogram (EOG) channels were identified as eye-movement-related artifacts and removed, preserving underlying neural activity.

3. Normalization: Z-score normalization was performed on each EEG channel:

(3) $$X_{\mathrm{norm}}=\frac{X-\mu }{\sigma }$$

with *X* representing the unprocessed EEG signal for a channel, μ its mean, and σ its standard deviation across the entire recording. Normalization ensures similar amplitude distributions across channels, preventing dominance by high-amplitude channels in subsequent steps.

#### 2.2.2. EMG Preprocessing

The EMG signal, reflecting muscle activation patterns from the middle deltoid (primarily indicating shoulder abduction level), was chosen as the ground truth for the regression task in this study, rather than direct kinematic measurements (e.g., from inertial measurement units (IMUs)). While IMUs provide a direct measure of limb posture and angle, the decision to target EMG was driven by several key considerations related to the study’s neurophysiological focus and its potential application in neuroprosthetics. Our primary aim was to decode the peripheral neuromuscular activation patterns (EMG) that arise directly from central cortical motor commands (EEG). This approach targets a signal that is physiologically closer to the originating cortical commands within the brain–muscle pathway compared to decoding kinematics, which represent the final outcome of this process. Furthermore, this choice holds direct relevance for neuroprosthetic control. Many advanced myoelectric prostheses are designed to interpret EMG patterns from residual muscles [21]. By successfully regressing EMG from EEG, this study aims to generate physiologically-inspired substitute control signals that could potentially interface directly with such existing hardware, offering a viable control strategy for individuals lacking sufficient residual muscle activity. To facilitate this, raw EMG was preprocessed as follows:

1. Filtering: Raw EMG underwent bandpass filtering (fourth-order zero-phase Butterworth, 40–95 Hz) to isolate muscle contraction frequencies, reducing low-frequency artifacts and high-frequency noise.

2. Rectification: The filtered EMG signal was full-wave rectified (absolute value), yielding a unidirectional signal reflecting muscle activation magnitude.

3. Smoothing: To derive a continuous EMG amplitude envelope, a fourth-order zero-phase Butterworth low-pass filter (2 Hz cutoff) was applied to the rectified signal.

(4) $$EMG_{smoothed}=\mathrm{filtfilt}(b_{smooth},a_{smooth},|\mathrm{filtfilt}\left(b_{emg},a_{emg},EMG_{Raw}\right)|)$$

where $b_{emg}$, $a_{emg}$ are coefficients for the 40–95 Hz bandpass filter, and $b_{smooth}$, $a_{smooth}$ for the 2 Hz low-pass filter. This yields a signal reflecting overall muscle activation, suitable as the regression target.

4. Normalization: The EMG data were z-score normalized.

### 2.3. Time-Frequency Visualization

Time-frequency representations (TFRs) were computed from C3, Cz, and C4 EEG channels to assess movement-related neural oscillations. EEG data were epoched relative to movement onset, and TFRs were generated using the pseudo Wigner–Ville distribution (PWVD) via MATLAB’s Time-Frequency Toolbox tfrrpwv function (Hanning window, 1024-point FFT).

Power spectra were baseline-corrected using the 1-s pre-movement period. Each time-frequency point was expressed in decibels relative to baseline power:

(5) $$P_{\mathrm{dB}}\left(t,f\right)=10\cdot \log_{10}\left(\frac{P(t,f)}{\mathrm{BaselinePower}(f)}\right)$$

Non-finite values were replaced with the minimum finite TFR value to ensure robust averaging.

**Figure 3:**
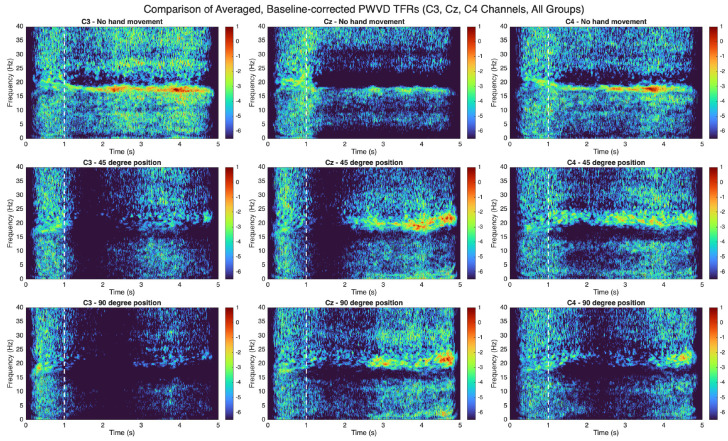
Each panel displays the average time-frequency representation (TFR) for one electrode (C3, Cz, or C4) across three hand movement conditions (no movement, 45°, 90° abduction). Power at each frequency was baseline-corrected using the 1-s pre-movement period and converted to decibels. The white dashed line indicates movement onset.

Baseline-corrected TFRs were averaged per hand angle group (low, middle, high abduction level). Group TFRs used a common color axis for direct comparison. Optimal color limits (cmin, cmax) were automatically selected to enhance visibility of group differences, especially in the 8–30 Hz, 0–3 s post-movement onset region of interest. This involved grid-searching candidate color limits (2nd to 98th percentile of pooled TFR values in this region), truncating TFR values, and computing a separability score (Fisher-like criterion: sum of absolute pairwise mean differences divided by pooled standard deviation). The color scale maximizing this score was chosen for Figure 3. The vertical white dashed line in each panel marks movement onset (end of the baseline period).

### 2.4. Feature Extraction

FBCSP and PLV were chosen as complementary approaches for amplitude-based and phase-based information extraction from EEG.

#### 2.4.1. Filter Bank Common Spatial Patterns

**Figure 4:**
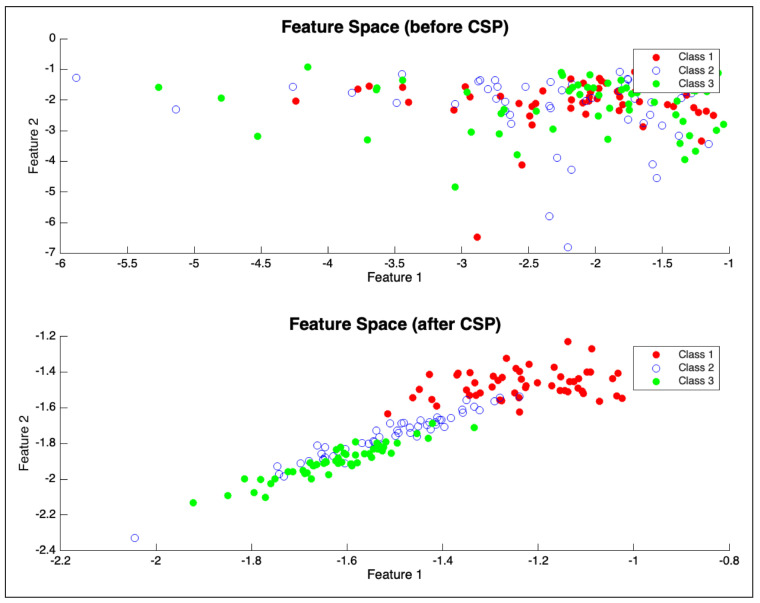
Schematic for the feature extraction process.

FBCSP derives spatial characteristics from EEG during motor tasks. It utilizes spatial organization across frequency ranges to improve differentiation between movement patterns. Steps performed:

1. Frequency Decomposition: EEG data were segmented into eight frequency bands via second-order Butterworth bandpass filters (0.5–4 Hz (delta), 4–8 Hz (theta), 8–12 Hz (alpha/mu), 12–16 Hz, 16–20 Hz, 20–24 Hz, 24–28 Hz, 28–32 Hz (beta)), covering neural oscillations relevant to motor control.

2. Common Spatial Patterns: The CSP algorithm [25] was applied to each band. For this regression task, CSP filters were derived by contrasting EEG data from movement periods against rest periods, aiming to find spatial filters that maximize variance related to motor activity. As shown in Figure 4, this approach effectively highlights distinctions between EEG patterns corresponding to different motor states.

(6) $$W_{\mathrm{CSP}}^{\left(f\right)}=\mathrm{MulticlassCSP}\left(filtered\_train\_data,cspn\right)$$

where filtered_train_data is EEG data for the *f*-th band (labeled for movement vs. rest), $W_{\mathrm{CSP}}^{\left(f\right)}$ are the CSP spatial filters, and ‘cspn’ = 10 (10 CSP components per band), chosen empirically.

3. Log-Variance Features: Log-variance of spatially filtered signals was computed. Variance reflects signal power; logarithm transforms data towards a Gaussian distribution.

(7) $${\mathrm{Feature}}_{\mathrm{FBCSP}}^{\left(f\right)}=\log_{10}\left(\frac{\mathrm{Var}(y^{\prime })}{\mathrm{sum}(\mathrm{Var}\left(y^{\prime }\right))}\right)$$

where $y=W_{\mathrm{CSP}}^{{\left(f\right)}^{\prime }}*filtered\_trial^{\prime }$, the spatially filtered EEG for a trial.

#### 2.4.2. Phase-Locking Value

While FBCSP extracts amplitude-based features (signal power), PLV measures functional connectivity via phase difference consistency between EEG signals from different electrodes. PLV is sensitive to neural oscillation synchronization, crucial for coordinating brain activity during motor tasks. PLV is:

(8) $$\mathrm{PLV}{\left(f\right)}_{xy}=\left|\frac{1}{N}\sum_{t=1}^{N}e^{j(\phi_{x}\left(t,f\right)-\phi_{y}\left(t,f\right))}\right|$$

where $\phi_{x}\left(t,f\right)$ and $\phi_{y}\left(t,f\right)$ are instantaneous phases of channels x,y at frequency *f*, and *N* is samples in the analysis window. PLV = 1 indicates perfect phase synchronization; PLV = 0 indicates no consistent phase relationship.

Instantaneous phase of each EEG channel was obtained via Hilbert transform:

(9) ϕ(t,f)=angle(hilbert(signal))

where ‘signal’ is bandpass-filtered EEG for a channel and frequency band. ‘angle’ extracts the phase angle.

To address potential time delays in inter-regional brain interactions, PLV was computed for simultaneous signals and six time lags: 0, 125, 250, 375, 500, and 625 ms. This allows capturing delayed interactions relevant for motor control dynamics (e.g., motor planning preceding execution). PLV was computed for all EEG channel pairs (496) and for each of the eight FBCSP frequency bands and six time lags.

### 2.5. Feature Selection

Combining FBCSP and PLV features resulted in a high-dimensional space, risking overfitting. The ReliefF algorithm [26] was used for dimensionality reduction and feature selection. ReliefF, a filter-based method, evaluates feature importance by assessing their ability to distinguish between instances with similar target EMG values (near hits) and those with different target EMG values (near misses), making it effective for regression tasks.

The ReliefF algorithm iteratively estimates feature weights. For each randomly selected instance, it identifies its nearest neighbors in the target variable space (EMG amplitude). The weights for each feature are updated based on its ability to differentiate the instance from its neighbors with different target values versus neighbors with similar target values. The score for feature $f_{i}$ is accumulated based on these differences.

The ReliefF algorithm assigns higher weights to features that provide more relevant information for the regression task. The top 50 features, ranked by their ReliefF scores, were selected as inputs to the neural network model. This number was empirically chosen to balance model performance with complexity. This data-driven approach identifies informative features for the specific task and dataset without prior assumptions.

#### Feature Importance Overview

**Figure 5:**
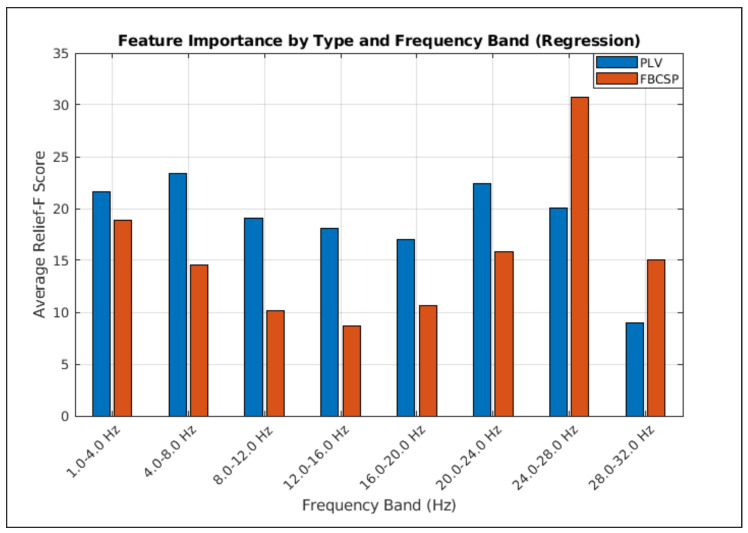
The bar plot displays the average ReliefF score for phase-locking value (PLV) and FBCSP features across eight frequency bands. Higher ReliefF scores indicate greater feature importance for the regression model.

Figure 5 illustrates average ReliefF scores, indicating the relative importance of PLV and FBCSP features across eight frequency bands for predicting arm movement. PLV features generally show higher importance than FBCSP features across most bands, suggesting a crucial role for phase synchronization.

Specifically, delta (0.5–4.0 Hz), theta (4.0–8.0 Hz), alpha/mu (8.0–12.0 Hz), and parts of the beta band (20.0–24.0 Hz and 24.0-28.0 Hz) appear particularly informative. While PLV is prominent in lower frequencies and the 20.0–24.0 Hz beta band, the 24.0–28.0 Hz beta band shows a notable contribution from FBCSP. Conversely, 12.0–16.0 Hz, 16.0–20.0 Hz, and 28.0–32.0 Hz bands demonstrate comparatively lower importance. These results suggest the value of considering both amplitude and phase-based features from specific frequency bands.

### 2.6. Ablation Study Design

To evaluate FBCSP and PLV feature contributions, an ablation study compared three feature set conditions:

1. FBCSP_only: Only FBCSP features (8 bands, 10 CSP components/band). After z-score normalization, ReliefF selected informative FBCSP features for the neural network.
2. PLV_only: Only PLV features (496 channel pairs, 8 bands, 6 time lags). After z-score normalization, ReliefF selected PLV features for the neural network.
3. Combined FBCSP and PLV (FBCSP_and_PLV): Both FBCSP and PLV features. Features were separately z-score normalized. ReliefF selected from the combined set for the neural network.

All conditions used the same feedforward neural network architecture and 6-fold cross-validation (Section 2.7 and Section 2.8). Performance metrics (RMSE, $R^{2}$, Pearson correlation) were calculated per participant and condition. Statistical comparisons used a Friedman test, then post-hoc Wilcoxon signed-rank tests with Bonferroni correction.

### 2.7. Neural Network Model

A feedforward neural network captured the non-linear mapping between EEG features and EMG amplitude. Specifically, a regression network was implemented using the ‘fitrnet’ function in MATLAB. This function was chosen for its ability to effectively model potentially complex, non-linear relationships between the high-dimensional fused EEG features (FBCSP and PLV) and the continuous EMG target. Furthermore, its built-in L2 regularization (detailed below) is crucial for preventing overfitting, particularly given the number of features relative to the sample size, thereby promoting better generalization of the decoding model.

Architecture: Two hidden layers: 90 neurons (first), 10 neurons (second). This structure was determined empirically, balancing complexity and overfitting risk.

Activation Function: Sigmoid activation for both hidden layers, introducing non-linearity.

Regularization: L2 regularization mitigated overfitting, with λ automatically tuned to $2.2135\times 10^{-7}$ by MATLAB.

Input: Selected features (FBCSP, PLV, or combined) from training data.

Output: Single value representing predicted EMG amplitude (z-score normalized pre-training).

Training: A 6-fold cross-validation strategy was employed. Data were partitioned into six folds. In each iteration, the model trained on five folds and tested on the remaining one.

### 2.8. Comparison with End-to-End Deep-Learning Models

For a comprehensive benchmark, two end-to-end deep-learning models were evaluated: a long short-term memory (LSTM) network and an ICNN-inspired convolutional neural network (CNN). Both regressed EMG amplitude directly from EEG data. For these E2E models, the 400 Hz preprocessed EEG data (Section 2.2.1) was further downsampled to 200 Hz using a decimation function with an anti-aliasing filter. The input consisted of 1-s segments of this 200 Hz EEG (32 channels, 0.18–40 Hz bandpass filtered) and the corresponding normalized EMG amplitude target. Models were trained using identical data splits and evaluation metrics as our proposed approach.

The LSTM captured temporal dependencies (LSTM layer, dropout, fully connected regression layer). The ICNN-inspired model used temporal/spatial convolutional layers, multi-scale feature branches, and a final regression output. Both used 6-fold cross-validation. RMSE, $R^{2}$, and Pearson correlation (R) were calculated per participant. Full architectural details, hyperparameters, and training procedures are in Supplementary Materials.

### 2.9. Model Evaluation

Neural network performance was assessed using three standard metrics, calculated per cross-validation fold and averaged across folds and participants.

#### 2.9.1. Pearson Correlation Coefficient (R)

Measures linear relationship between predicted and actual EMG amplitudes:

(10) $$R=\frac{\mathrm{cov}(Y_{\mathrm{pred}},Y_{\mathrm{true}})}{\sigma_{Y_{\mathrm{pred}}}\sigma_{Y_{\mathrm{true}}}}$$

where $\mathrm{cov}(Y_{\mathrm{pred}},Y_{\mathrm{true}})$ is covariance, $\sigma_{Y_{\mathrm{pred}}}$ and $\sigma_{Y_{\mathrm{true}}}$ are standard deviations.

#### 2.9.2. Root Mean Squared Error (RMSE)

Quantifies average magnitude of discrepancies between predicted and actual EMG:

(11) $$\mathrm{RMSE}=\sqrt{\frac{1}{N}\sum_{i=1}^{N}{\left(Y_{\mathrm{pred}}\left(i\right)-Y_{\mathrm{true}}\left(i\right)\right)}^{2}}$$

where *N* is number of samples.

#### 2.9.3. Coefficient of Determination ($R^{2}$)

Proportion of EMG signal variance explained by the model:

(12) $$R^{2}=1-\frac{\sum_{i=1}^{N}{\left(Y_{\mathrm{pred}}\left(i\right)-Y_{\mathrm{true}}\left(i\right)\right)}^{2}}{\sum_{i=1}^{N}{\left(Y_{\mathrm{true}}\left(i\right)-\bar{Y_{\mathrm{true}}}\right)}^{2}}$$

where $\bar{Y_{\mathrm{true}}}$ is mean of true EMG amplitudes.

### 2.10. Subjects and Equipment

**Figure 6:**
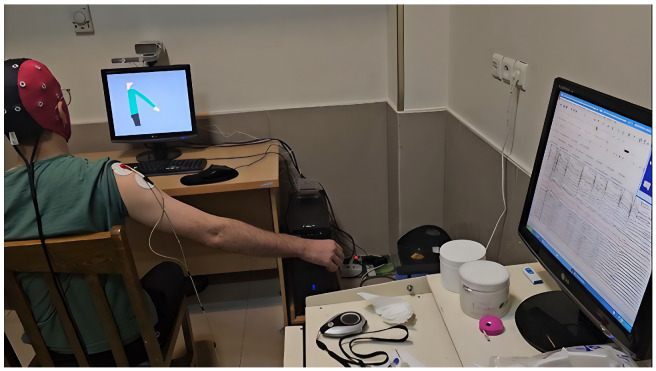
Subject and signal acquisition setup.

This study used a semi-experimental design to evaluate decoding arm movement parameters (Figure 6) using combined EEG features. Ten healthy right-handed individuals (no motor disabilities) participated (Table 1). Participants (age range 25–30, mean 26.9 ± 1.37 years) were selected via convenience sampling. Demographics: 70% male, 30% female; 80% master’s degree, 20% bachelor’s; 40% prior EEG experience. Occupations: 60% employed, 20% students, 20% unspecified. All were right-handed (Edinburgh Handedness Inventory [27]).

#### 2.10.1. Inclusion and Exclusion Criteria

Eligible participants: healthy, 25–30 years, no history of neurological disorders, acute psychological conditions, or substance addictions. Exclusion: consumption of caffeine, chocolate, smoking, or other tobacco products within 24 h pre-experiment to minimize confounding effects.

#### 2.10.2. EEG Recording Setup

**Figure 7:**
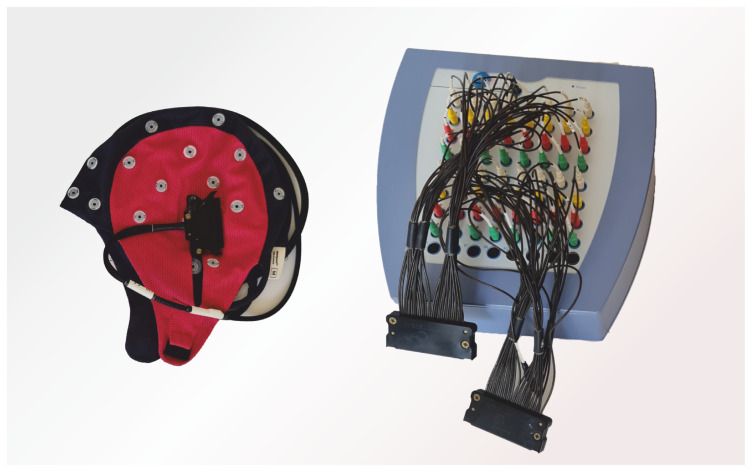
REFA amplifier and 32 electrode Waveguard EEG cap used in this study.

EEG signals were recorded using an ANT neuro REFA amplifier (Figure 6 and Figure 7) with 32 channels (international 10–20 standard [28]). A WaveGuard 32-electrode cap (sized per participant) secured electrodes. Cz was reference, POz was ground [29].

#### 2.10.3. EMG Recording Setup

EMG signals were recorded via a single bipolar channel (surface electrodes over middle deltoid tendon, primarily reflecting shoulder abduction) using a high-fidelity EMG amplifier. The amplifier filtered signals through a rectifier circuit, ensuring analog output voltage varied proportionally with muscle contraction intensity. During calibration, participants performed arm lifts at designated abduction levels to establish baseline EMG for different movement states.

#### 2.10.4. Data Acquisition Software and Hardware

EEG and EMG data were synchronized and recorded using MATLAB-based software for real-time acquisition and preprocessing. EEG data, initially sampled at 512 Hz, were resampled to 400 Hz for the feature-based analysis.

## 3. Results

### 3.1. Performance of Combined Features for EMG Decoding

This research evaluated an integrated methodology, combining FBCSP for amplitude and PLV for phase connectivity, to decode arm movement (represented by EMG signals from the middle deltoid) from EEG. Data from ten participants were evaluated using a 6-fold cross-validation procedure.

### 3.2. Ablation Study Results

An ablation study investigated individual and combined contributions of FBCSP and PLV features. Average performance for FBCSP_only, PLV_only, and combined FBCSP_and_PLV conditions are in Table 2.

The FBCSP_and_PLV condition achieved the best performance (lowest RMSE, highest $R^{2}$ and Pearson R). A Friedman test revealed significant differences among conditions for RMSE ($\chi^{2}\left(2\right)=15.800,\;p=0.0004$), $R^{2}$ ($\chi^{2}\left(2\right)=15.800,\;p=0.0004$), and Pearson R ($\chi^{2}\left(2\right)=16.800,\;p=0.0002$). Post-hoc Wilcoxon signed-rank tests (Bonferroni corrected) showed FBCSP_and_PLV significantly outperformed FBCSP_only (RMSE: p=0.002; $R^{2}$: p=0.002; Pearson R: p=0.002) and PLV_only (RMSE: p=0.002; $R^{2}$: p=0.002; Pearson R: p=0.002). No significant difference in RMSE (p=0.7695) or $R^{2}$ (p=0.7695) between FBCSP_only and PLV_only was found, though FBCSP_only tended towards higher Pearson R (p=0.0645). These findings highlight the benefit of integrating FBCSP and PLV features.

**Figure 8:**
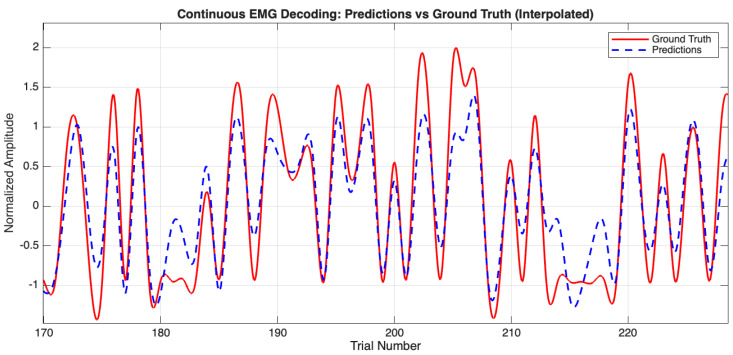
EEG decoding: predicted vs. ground truth amplitudes for Participant 3. The red line represents the actual EMG amplitudes, while the dashed blue line denotes the model’s predictions, showcasing the close tracking achieved.

The neural network model, trained on the combined FBCSP and PLV features, demonstrated notable decoding capabilities (Table 3). For instance, Participant 3 achieved R = 0.894 and $R^{2}$ = 0.784, indicating the model captured nearly 80% of EMG signal variance. This performance, supported by Figure 8, highlights the efficacy of integrating amplitude and connectivity features. Conversely, results for Participant 8 (R = 0.666, R^2^ = 0.430) illustrate inter-subject variability. On average, the model achieved R = 0.829 ± 0.077, R^2^ = 0.675 ± 0.126, and RMSE = 0.579 ± 0.098, underscoring the general value of our feature fusion.

To situate these findings within the broader landscape of EEG-based movement decoding, Table 4 provides a comparison with recent studies, sectioned by the primary decoding task to facilitate a more nuanced interpretation. This study (Table 4, Section A, Entry 2), focusing on EEG-to-EMG estimation for arm movements, achieved results that are directly comparable to, and slightly exceed, the R≈0.78 reported by Liang et al. (2020) [22] for shoulder EMG estimation, despite their use of fewer EEG channels. This suggests that our approach, combining FBCSP and PLV features with ReliefF selection and a neural network regressor, offers a potent method for mapping cortical activity to peripheral muscle activation.

When considering the broader context of continuous kinematic decoding from EEG (Table 4, Section B), it is important to acknowledge the difference in the decoded variables (EMG vs. direct kinematics like position or velocity). Studies in this category, such as Mondini et al. (2020) [11] decoding robotic arm kinematics (R≈0.32–0.5), Borra et al. (2023) [12] decoding 2D hand kinematics (R≈0.4–0.5), and Hosseini and Shalchyan (2023) [18] decoding center-out trajectories (R≈0.37–0.54), generally report correlation coefficients in a lower to moderate range in accordance with task difficulty. While a direct performance comparison is nuanced due to the distinct nature of the targets—EMG potentially being more directly linked to cortical motor commands than endpoint kinematics—our findings indicate a strong predictive capability that is competitive within the overall field of continuous EEG-based motor decoding.

Section C of Table 4 includes studies on discrete motor intent classification (e.g., Kuo et al. (2012) [30]; Kaviri and Vinjamuri (2025) [31]), reporting accuracy metrics. These are included for breadth but are fundamentally different from our regression approach and not directly comparable by performance metrics. Our study enhances continuous decoding by fusing amplitude and connectivity for EMG prediction.

**Table 4:** Comparison of recent EEG movement decoding studies, grouped by task type.

#	Study (Year)	Task / Target	Subj.	EEG System	EEG Features	Results (Metrics)
Section A: EEG-to-EMG Estimation (Kinesthetic Information—Upper Limb)						
1	Liang et al. (2020) [22]	Shoulder EMG est. from EEG (flex/ext)	3	7-ch g.GAMMAsys, Deltoid EMG	ICA+STFT+PCA of EEG	R=0.78±0.037
2	This study	Arm movement angles (via EMG from EEG)	10	32-ch ANT REFA; Deltoid EMG	FBCSP + PLV, ReliefF, NN	R=0.829±0.077, $R^{2}=0.675$
Section B: Continuous Kinematic Decoding (Upper Limb)						
3	Mondini et al. (2020) [11]	2D pursuit, online robotic arm (Pos, Vel)	10	64-ch BrainAmp, LeapMotion	Low-freq EEG (0.18–1.5 Hz)	R≈0.32 (on), R≈0.5 (off)
4	Sosnik & Zheng (2021) [32]	3D limb vel. (hand, elbow, shoulder)	9	61-ch, Kinect 3D	sLORETA (PTS, BTS), SCPs	R=0.36 (act), R=0.18 (img)
5	Robinson et al. (2021) [19]	Hand speed (class.), 1D pos/speed (regr.)	21	Emotiv EPOC (8 eff. ch.)	Sub-band low-freq, FBCSP	R≈0.38–0.40 (pos)
6	Hosseini & Shalchyan (2022) [17]	2D center-out hand pos. (Px, Py)	7	63-ch g.HIamp, LeapMotion	PLV, MSC (phase conn.)	$R_{\mathrm{PLV}}=0.43$, $R_{\mathrm{MSC}}=0.42$
7	Borra et al. (2023) [12]	2D hand pos/vel pursuit	13	53-ch (10-10), LeapMotion	Raw EEG into ICNN	$R_{\mathrm{pos}}\approx 0.5$, $R_{\mathrm{vel}}\approx 0.4$
8	Hosseini & Shalchyan (2023) [18]	Center-out (4-axis) + random targets	9	63-ch g.HIamp, LeapMotion	CSP (pre-mov) + envelope	R=0.54 (princ.), 0.37 (rand.)
Section C: Discrete Motor Intent Classification (Upper Limb)						
9	Kuo et al. (2012) [30]	Pre-movement direction (L/R/F)	8	128-ch HydroCel (EGI)	Mean ampl. (PPC)	93.9% acc. (L/R)
10	Kaviri & Vinjamuri (2025) [31]	4-class MI, 6-class ME (grasp)	9/10	22/32-ch, CyberGlove	Source loc. + ICA + PSD	MI: 99.15%, ME: 90.83% (acc.)

### 3.3. Frequency-Specific PLV Connectivity Patterns

**Figure 9:**
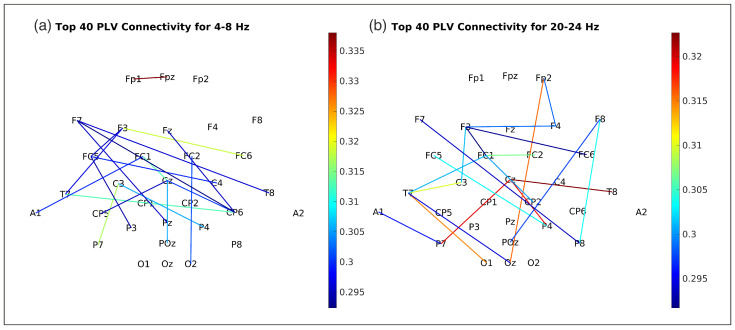
The figure displays the 40 strongest PLV connections for (**a**) 4–8 Hz, and (**b**) 20–24 Hz. Each node represents an EEG electrode, labeled according to the 10–20 system. Lines connecting nodes represent significant PLV connections, with line color indicating the strength of the connection (warmer colors represent stronger PLV). The spatial distribution of these connections provides insights into the functional connectivity patterns within each frequency band.

Figure 9 shows the top 40 PLV connections. Delta band (0.5–4 Hz, panel a) shows a predominantly fronto-central and centro-parietal network, suggesting large-scale coordination. Theta band (4–8 Hz, panel b) reveals similar patterns, with potential frontal and parietal interaction emphasis, hinting at cognitive control. The 20–24 Hz beta band (panel c) displays a more distributed network (frontal, central, parietal), possibly reflecting complex sensorimotor integration. Color intensity (connection strength) highlights dynamic inter-regional interplay across frequency bands. Figure 3 presents TFRs across the C3, Cz, and C4 electrodes during three movement conditions. The no-movement condition (top row) shows minimal oscillatory changes, serving as a control. The 45° abduction condition (middle row) displays moderate beta-band event-related desynchronization (ERD) around movement onset, with C3 showing the most pronounced contralateral motor cortex activation. The 90° abduction condition (bottom row) exhibits strong beta ERD across all electrodes, followed by post-movement beta event-related synchronization (ERS), indicating increased motor cortex engagement with higher movement demands. C4 (ipsilateral) generally shows weaker modulation compared to C3 (contralateral), while Cz demonstrates intermediate responses. The consistent color scale across panels highlights the systematic modulation of sensorimotor rhythms with movement intensity and validates their importance for motor decoding applications.

### 3.4. Comparison with End-to-End Deep Learning Models

To further evaluate our proposed feature-fusion method (FBCSP + PLV + NN), we compared it against an LSTM network and an ICNN-inspired CNN. These models were trained and tested on the identical dataset and cross-validation folds (Section 2.8, Supplementary Materials). Average performance metrics are in Table 5.

Table 5 indicates the LSTM model achieved the highest performance (mean Pearson R = 0.934±0.020, $R^{2}$ = 0.874±0.037, RMSE = 0.423±0.064), notably higher than our feature-based method (Pearson R = 0.829±0.077). The ICNN model also showed strong decoding (mean Pearson R = 0.860±0.070, $R^{2}$ = 0.741±0.103), superior to our method in R and $R^{2}$, though its RMSE (0.601±0.116) was slightly higher than our method’s (0.579±0.098). Overall, end-to-end deep learning, particularly LSTMs, effectively decoded EMG from EEG in this study, reaching higher predictive accuracy than the traditional feature engineering pipeline. This superior accuracy is discussed alongside interpretability, computational complexity, and BCI system insights in Section 4.6. Further LSTM/ICNN performance details are in Supplementary Materials.

## 4. Discussion

### 4.1. Movement Decoding Through Feature Fusion

This study demonstrates that combining amplitude-based (FBCSP) and phase-based (PLV) features effectively decodes arm movements from non-invasive EEG. Our neural network, with data-driven feature selection, achieved an average Pearson correlation R = 0.829±0.077, $R^{2}$ = 0.675±0.126, and RMSE = 0.579±0.098, outperforming single-feature approaches. These results validate our hypothesis that integrating complementary neural signatures enhances decoding accuracy.

### 4.2. The Role of Phase Synchronization in Motor Decoding

PLV features were dominant contributors, especially in delta (0.5–4 Hz), theta (4–8 Hz), and beta (20–24 Hz) bands (Figure 5), aligning with known neurodynamics. Delta-phase synchronization likely facilitates large-scale cortical integration in movement planning [33], while theta-band PLV may coordinate sensorimotor feedback [34]. Beta-band PLV (20–24 Hz) prominence suggests its role in sustaining motor output via corticomuscular coherence [35]. PLV’s sensitivity to delayed interactions (via time-lags) captured directional connectivity (e.g., frontal-to-parietal flow during movement initiation, Figure 9), inaccessible to amplitude-only methods.

### 4.3. Complementary Contributions of Amplitude and Connectivity Features

While PLV dominated lower frequencies, FBCSP provided critical beta-band (24–28 Hz) amplitude information (Figure 5), consistent with motor-related beta desynchronization [10]. This synergy suggests distinct yet complementary mechanisms: PLV encodes distributed network dynamics, FBCSP captures localized power changes. ReliefF prioritized features across this spectrum, with a substantial portion of selected features involving PLV connections between frontoparietal nodes, underscoring inter-regional communication importance.

### 4.4. Interpreting Feature Importance Across Frequency Bands

The ReliefF feature importance scores (Figure 5) indicate varying contributions from different frequency bands. PLV features from delta (0.5–4 Hz) and theta (4–8 Hz) bands showed high importance, consistent with roles in large-scale cortical integration during motor planning [33] and sensorimotor feedback coordination [34], respectively. Alpha/mu band (8–12 Hz) PLV also contributed significantly, potentially reflecting inhibitory processes gating sensorimotor integration [36].

In the beta range, PLV features in the 20–24 Hz sub-band were highly ranked, aligning with its role in sustained motor output and corticomuscular coherence [35]. Concurrently, FBCSP features from the 24–28 Hz beta sub-band also showed high importance, likely capturing motor-related beta desynchronization [10]. This highlights that both phase synchronization and amplitude modulations within specific beta sub-bands are critical for decoding. The relatively lower importance of features from mid-beta (e.g., 12–20 Hz) and high-beta (28–32 Hz) in this specific task might suggest task-dependent engagement of these narrower frequency ranges or limitations in non-invasively capturing their finer spatial patterns due to volume conduction. These findings emphasize that integrating information from specific low-frequency (delta, theta, alpha) connectivity and distinct beta-band amplitude and connectivity features can enhance decoding frameworks.

### 4.5. Rationale for Incorporating Phase-Based Connectivity Features

Phase-based connectivity features (PLV) were included as they offer distinct, complementary information to amplitude-based measures. PLV quantifies phase difference consistency between signals over time. Unlike amplitude/power (energy in frequency bands), phase features capture temporal synchronization of neural oscillations between brain regions. Phase and power are largely independent EEG aspects [37]; combining them provides a more complete characterization of neural dynamics. Amplitude features were extracted via FBCSP (power changes). PLV features were computed for EEG channel pairs and frequency bands (functional connectivity). Integrating PLV and FBCSP features in a neural network leverages local signal modulations and distributed synchronization. Our ablation study (Table 2) confirmed that combining these features yields better decoding than either alone. Thus, phase-based connectivity inclusion enhances neural information richness and robustness for EMG decoding from EEG, supporting more effective BCIs.

### 4.6. Comparison with End-to-End Architectures and Methodological Considerations

Our comparative analysis (Section 3.4, Table 5) showed that an LSTM network achieved superior EMG decoding accuracy (mean Pearson R = 0.934±0.020) on this dataset compared to our FBCSP + PLV feature-fusion method (R = 0.829±0.077) and an ICNN-inspired model. This highlights deep networks’ capacity to discern complex EEG patterns. However, pursuing maximal accuracy involves trade-offs:

1. Interpretability and Neurophysiological Insight: Our feature-based method is more interpretable. Engineered FBCSP/PLV features allow insights into contributing dynamics (e.g., specific frequency band roles, inter-regional synchronization; Figure 5, Figure 9). End-to-end models are often “black boxes”, making it hard to discern the neurophysiological basis of learned features without substantial post-hoc analysis, limiting direct neuroscientific knowledge extraction and failure understanding.
2. Computational Demands and Real-Time Viability: Training complex deep-learning models (e.g., LSTMs) is computationally intensive, often needing specialized hardware (GPUs) and extensive tuning. Deploying large models in resource-constrained, real-time BCIs can also be challenging. Our neural network with selected features is likely less computationally demanding at inference.

In summary, while LSTMs showed superior accuracy here, BCI modeling choice depends on application goals. If maximal accuracy is paramount and resources allow, deep learning is compelling. For applications prioritizing neural correlate understanding, model transparency, or real-time feasibility with limited resources, feature-based methods like ours retain value. Our FBCSP + PLV approach offers a robust, interpretable framework for EEG-based EMG decoding. Future work could explore hybrid models or more inherently interpretable deep-learning architectures. Applying advanced interpretability techniques to the successful LSTM could also reveal exploited EEG patterns. Further details are in Supplementary Materials.

### 4.7. Advantages and Limitations

**Figure 10:**
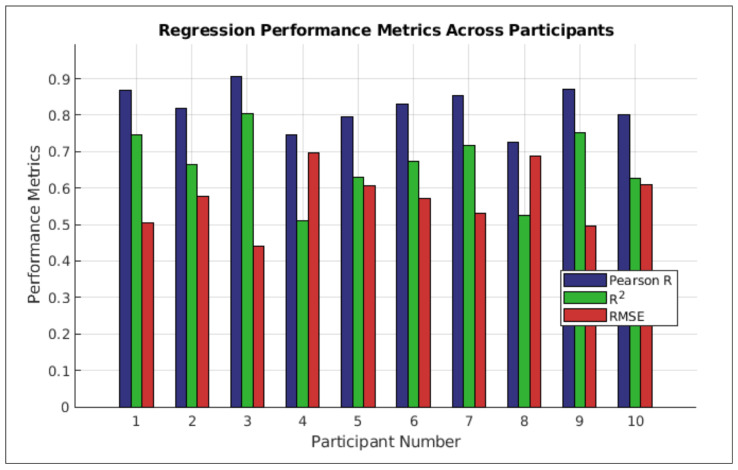
Regression performance metrics across subjects. Bar graphs representing the Pearson correlation coefficient (*R*), coefficient of determination ($R^{2}$), and root mean square error (RMSE) for each participant.

Our study decodes EMG from non-invasive EEG using a neural network integrating FBCSP (amplitude) and PLV (phase) features. The achieved accuracy (mean Pearson R = 0.829±0.077; Table 4, Entry 2 and Figure 10) with non-invasive EEG offers a practical alternative to invasive methods, potentially broadening BCI application by mitigating clinical risks.

The core of our methodology is the fusion of complementary neural information streams. It combines FBCSP analysis (amplitude modulations) with PLV analysis (phase synchronization for functional connectivity). While other studies (Table 4, Section B) have used PLV (e.g., Hosseini and Shalchyan, 2022 [17]) or FBCSP (e.g., Robinson et al., 2021 [19]) for kinematic decoding, our work (Entry 2, Section A) distinctively applies this amplitude-phase integration for EMG estimation. The results from our ablation study (Table 2) further reinforce this, demonstrating that a combined feature set outperformed using either FBCSP features alone or PLV features alone for this dataset. This integrated approach aims to provide a more comprehensive representation of the neural processes underlying muscle engagement compared to methods focusing on a single feature type or decoding kinematic variables. The primary advantage of this feature-engineering approach, particularly when contrasted with more accurate but less transparent end-to-end models (Section 3.4), lies in its enhanced interpretability, allowing for insights into the contributing neurophysiological mechanisms (Figure 5 and Figure 9). While our neural network itself is a complex decoding model, the interpretability stems from the selection and analysis of physiologically-motivated input features. This is an important consideration, as direct interpretation of weights from multivariate decoders can be misleading if not properly transformed or contextualized, as highlighted by [38].

However, several limitations must be acknowledged. Firstly, while our proposed method demonstrates robust performance, the comparative analysis revealed that an end-to-end LSTM model achieved superior decoding accuracy on this specific dataset (Table 5). This suggests that for applications where maximal predictive power is the primary goal and the “black box” nature of the model is acceptable, alternative architectures might be more suitable. Secondly, inherent EEG limitations (lower spatial resolution, higher artifact susceptibility vs. invasive recordings) persist. EEG-to-EMG decoding accuracy may differ from direct neural recordings. Furthermore, the findings, including those from the ablation study and the end-to-end model comparisons, are based on a relatively small sample of 10 participants. While statistical analyses indicated clear benefits for the combined feature approach and highlighted performance differences with deep-learning models within this cohort, the small sample size may limit statistical power to detect smaller effects, and further research with larger, more diverse participant groups is necessary to confirm the generalizability of these results and the robustness of the observed performance gaps.

A further consideration, critical to the ultimate clinical application of EEG-to-EMG decoding, is its utility for individuals with severely impaired or absent EMG activity. Our current study, by design, relied on healthy participants where EMG provided a direct ground truth for model training. For target users lacking such signals, direct application of this specific regression model is challenging. While using normative EMG data from healthy individuals as a reference is a conceptual possibility, significant inter-subject variability in both EEG motor patterns and EMG expressions for similar intended movements would likely necessitate highly sophisticated subject-specific adaptation or alternative approaches. Future research for this population might explore shifting the decoding target to discrete motor intentions, leveraging motor imagery paradigms, or employing user-in-the-loop adaptive learning and reinforcement learning strategies that do not strictly require concurrent ground-truth EMG from the user. These avenues, however, introduce their own complexities and represent substantial research challenges in themselves, including the development of robust calibration techniques and managing potential increases in user cognitive load.

Nevertheless, a key aspect of this work is the successful integration of distinct, physiologically-motivated feature domains from non-invasive recordings for muscle activity decoding. This approach contributes to the development of accessible and interpretable BCI systems for motor control, offering a valuable alternative, particularly when understanding the underlying neural dynamics is as important as decoding performance, a perspective that complements direct kinematic decoding strategies or purely accuracy-driven end-to-end methods.

## 5. Conclusions

This study investigated decoding hand movement-related EMG amplitudes from non-invasive EEG by integrating FBCSP (amplitude-based) and PLV (phase-based connectivity) features within a neural network. The approach yielded a mean Pearson correlation of 0.829±0.077 and RMSE of 0.579±0.098 across ten healthy participants (Table 4, Entry 2). These results suggest the potential of combining these complementary feature types for detailed movement-related decoding in non-invasive BCIs. Such findings may contribute to developing neuroprosthetic control systems, potentially improving solutions for individuals with severe motor disabilities by aiming for a more direct link to muscle engagement. 

## Figures and Tables

**Table 1:** Demographic characteristics of participants.

Participant	Age	Education	Identification	Occupation
1	26	Master’s	Male	Unemployed
2	27	Master’s	Male	Employed
3	28	Bachelor’s	Male	Employed
4	27	Master’s	Female	Employed
5	26	Master’s	Female	Student
6	30	Master’s	Male	Unemployed
7	26	Master’s	Female	Employed
8	25	Bachelor’s	Male	Employed
9	27	Master’s	Male	Student
10	27	Master’s	Male	Employed

**Table 2:** Ablation study performance metrics across all subjects (mean ± SD).

Feature Set Condition	RMSE	$R^{2}$	Pearson R
FBCSP_only	0.818±0.156	0.307±0.259	0.688±0.093
PLV_only	0.836±0.087	0.292±0.143	0.601±0.094
FBCSP_and_PLV	0.579±0.098	0.675±0.126	0.829±0.077

**Table 3:** Aggregated regression performance metrics across all subjects (proposed combined feature method).

Participant	Mean RMSE	Mean $R^{2}$	Mean Pearson *R*
1	0.469	0.779	0.889
2	0.564	0.681	0.832
3	0.464	0.784	0.894
4	0.737	0.455	0.708
5	0.557	0.689	0.834
6	0.608	0.628	0.806
7	0.540	0.707	0.853
8	0.754	0.430	0.666
9	0.499	0.750	0.889
10	0.595	0.645	0.824
Average	0.579 ± 0.098	0.675 ± 0.126	0.829 ± 0.077

**Table 5:** Performance comparison with end-to-end deep learning models on the current dataset (mean ± SD across 10 subjects).

Model	RMSE	$R^{2}$	Pearson R
Proposed (FBCSP + PLV + NN)	0.579±0.098	0.675±0.126	0.829±0.077
LSTM	** 0.423±0.064 **	** 0.874±0.037 **	** 0.934±0.020 **
ICNN-inspired	0.601±0.116	0.741±0.103	0.860±0.070

## Data Availability

The datasets generated and analyzed during the current study are available from the corresponding author upon reasonable request.

## Supplementary Materials

The following supporting information can be downloaded at: https://www.mdpi.com/article/10.3390/bioengineering12060614/s1.

## Abbreviations

The following abbreviations are used in this manuscript:

BCI	Brain–computer interface
CSP	Common spatial patterns
CV	Cross-validation
ECoG	Electrocorticographic
EEG	Electroencephalography
EMG	Electromyography
EOG	Electrooculography
ERD	Event-related desynchronization
ERS	Event-related synchronization
FBCSP	Filter bank common spatial patterns
ICSS	Institute for Cognitive Science Studies
ICA	Independent component analysis
LFPs	Local field potentials
PLV	Phase-locking value
RMSE	Root mean square error
SRU	Shahid Rajaee Teacher Training University
SSVEP	Steady-state visual evoked potentials
